# Dynamic Subunit Exchange and the Regulation of Microtubule Assembly by the Stress Response Protein Human αB Crystallin

**DOI:** 10.1371/journal.pone.0011795

**Published:** 2010-07-26

**Authors:** Scott A. Houck, John I. Clark

**Affiliations:** 1 Department of Biological Structure, University of Washington, Seattle, Washington, United States of America; 2 Department of Ophthalmology, University of Washington, Seattle, Washington, United States of America; 3 Medicine Administration, University of North Carolina, Chapel Hill, North Carolina, United States of America; Universität Heidelberg, Germany

## Abstract

**Background:**

The small heat shock protein (sHSP), human αB crystallin, forms large, polydisperse complexes that modulate the tubulin-microtubule equilibrium using a dynamic mechanism that is poorly understood. The interactive sequences in αB crystallin for tubulin are surface exposed, and correspond to interactive sites for the formation of αB crystallin complexes.

**Methodology/Principal Findings:**

There is sequence homology between tubulin and the interactive domains in the β8-strand of the core domain and the C-terminal extension of αB crystallin. This study investigated the hypothesis that the formation of tubulin and αB crystallin quaternary structures was regulated through common interactive domains that alter the dynamics of their assembly. Size exclusion chromatography (SEC), SDS-PAGE, microtubule assembly assays, aggregation assays, multiple sequence alignment, and molecular modeling characterized the dynamic response of tubulin assembly to increasing concentrations of αB crystallin. Low molar ratios of αB crystallin∶tubulin were favorable for microtubule assembly and high molar ratios of αB crystallin∶tubulin were unfavorable for microtubule assembly. Interactions between αB crystallin and unassembled tubulin were observed using SEC and SDS-PAGE.

**Conclusions/Significance:**

Subunits of αB crystallin that exchange dynamically with the αB crystallin complex can interact with tubulin subunits to regulate the equilibrium between tubulin and microtubules.

## Introduction

A dynamic equilibrium between tubulin subunits and assembled microtubules is the mechanism for microtubule assembly *in vitro* and in mitosis/meiosis, intracellular transport, cell motility, and cell shape [1]. Microtubule assembly is GTP-dependent and carefully regulated by post-translational modification, ion concentrations, pH, calcium, phosphorylation, microtubule associated proteins (MAPs) and tubulin binding reagents [2], [3]. Hyperphosphorylation of the protein tau, a MAP linked with neurodegeneration, promotes destabilization of microtubules in Alzheimer's disease which makes MAPs promising targets for therapeutic approaches to Alzheimer's disease [4]. Anti-mitotic drugs that disrupt the dynamic equilibrium of microtubules can be used in the treatment of cancer [5]. The dynamic equilibrium is not limited to the mechanism for microtubule assembly. The self assembly of filament proteins including actin, viral coat proteins and spherical complexes of mammalian small heat shock proteins (sHSP) are dynamic equilibria suggesting the fundamental importance of dynamic equilibria in self assembly of numerous cellular macromolecules [6], [7], [8], [9], [10].

The dynamic equilibrium is a mechanism for assembly of heterogeneous macromolecular structures containing variable numbers of subunits. Microtubules can vary dramatically in length and form, from filaments to tubules to sheets [11]. Similarly the dynamic spherical complexes of αB crystallin, the archetype for sHSP, vary greatly in size, with the median size reported to be approximately 24 subunits [10], [12]. As with other dynamic molecular equilibria, the assembly of the αB crystallin complex is regulated by cofactors including Ca^2+^, ATP, and arginine-HCl [12], [13], [14]. The αB crystallin subunit consists of non-conserved N- and C-terminal domains and a highly conserved α crystallin core domain. All three structural domains contain interactive sequences for recognition, selection and solubilization of unfolding protein and for subunit-subunit interactions in the self assembly of the polydisperse complexes [15], [16], [17], [18]. The specific surface exposed sequences used for interactions with unfolding proteins overlap with sequences used for interactions between αB crystallin subunits, suggesting that the accessibility of the interactive sequences has functional significance. We propose that the overlap in interactive sequences for subunits of tubulin or αB crystallin is critical to a unique, dynamic mechanism for sHSP regulation of tubulin assembly.

An important function of αB crystallin is the stabilization of the assembly of microfilaments, intermediate filaments (IF) and microtubule networks [19], [20], [21], [22]. The interactive sequences in αB crystallin used for these functions have been identified using a variety of techniques, including pin arrays and mutagenesis [12], [15], [16], [22], [23]. Introduction of point mutations at the Arg120 in the interactive sequence of αB crystallin caused defective interactions with the IFs resulting in destabilized IF networks, cataract and desmin-related myopathy [24]. In response to cellular stress, αB crystallin was reported to bind actin microfilaments and aid in regulating actin dynamics in pinocytosis, thus preserving cell viability [25]. It is well established that αB crystallin has a regulatory effect on the dynamic assembly of microtubules [15], [26], [27], [28]. In cultured lens epithelial cells from α crystallin null mice, the microtubule length increased by about 2.5 fold [26]. This result suggested that concentrations of intracellular α crystallin as high as those found in the biological lens have an inhibitory effect on microtubule assembly in cells. *In vitro* assembly assays also have shown that concentrations of αB crystallin exceeding that of tubulin inhibited microtubule assembly [15], [27]. In separate reports αB crystallin was found to stabilize microtubules by promoting assembly or, in contrast, to prevent disassembly and aggregation [15], [29], [30], [31]. Consistent with the latter studies, αB crystallin expression increased in cells cultured in the presence of microtubule depolymerizing reagents, perhaps to assist with stabilization of the cytoskeleton [32], [33]. While the results of these studies could appear to be in conflict, the hypothesis tested in this report is that the formation of tubulin and αB crystallin quaternary structures can be regulated through common interactive domains that alter the dynamics of their assembly.

Previously, interactive sequences in human αB crystallin were identified using protein pin-arrays. Bioactive peptides based on the previously identified interactive sequences in human αB crystallin were synthesized and tested on microtubule assembly *in vitro* [15], [34]. The sequences _131_LTITSSLSSDGV_142_ and _156_ERTIPITRE_164_ in αB crystallin promoted microtubule assembly, and the sequence _113_FISREFHR_120_ inhibited microtubule assembly [15]. The sequences _131_LTITSSLSSDGV_142_ and _156_ERTIPITRE_164_, which promoted tubulin assembly, were sites of interactions between αB crystallin subunits during formation of αB crystallin complexes and the _113_FISREFHR_120_ sequence that inhibited tubulin assembly included surface exposed side chains that were not sites of subunit-subunit interactions [35], [36], [37], [38].

In the current study, a DAPI fluorescence assay was used to quantify the effects of selected molar ratios of αB crystallin∶tubulin on microtubule assembly and aggregation. Size-exclusion chromatography measured the size and polydispersity of large complexes formed between αB crystallin and unassembled tubulin. Sequence analysis found that microtubules have an interactive site for αB crystallin near an interface for assembly on the luminal side of the microtubule, in a similar interactive domain identified previously as taxol and tau binding sites [39]. The sequencing results were consistent with a common regulatory domain for the dynamic assembly of the αB crystallin complex and the dynamic assembly of microtubules [15]. The dependence of microtubule assembly on the molar ratio of αB crystallin∶tubulin was non-linear, appeared to be parabolic, and was characterized by an increase in microtubule assembly to a maximum at small molar ratios of αB crystallin∶tubulin, followed by a decrease in microtubule assembly at large molar ratios of αB crystallin∶tubulin. The inhibition of microtubule assembly at low and high molar ratios of αB crystallin∶tubulin is consistent with an unique dynamic mechanism for sHSP in the regulation of the self assembly of macromolecular structures including microtubules.

## Materials and Methods

### Purification of αB crystalline

αB crystallin was purified from E. coli as previously described [40]. *E coli* BL21 (DE3) cells (Stratagene) transformed with a human αB crystallin pET16b plasmid were grown for 12 hours at 37°C on an LB-agar plate containing carbenicillin. A single colony was isolated, added to 250mL starter culture of LB-broth+carbenicillin, and incubated for 12 hours at 37°C. 2.5mL of the starter culture was added to 12 flasks each containing 250mL of LB-broth+carbenicillin. Cells were grown at 37°C to optical density at 595nm >0.5. αB crystallin expression was induced with 0.25 mL of 1 M isopropyl-β-d-thiogalactopyranoside (IPTG) per flask. Three hours after induction, cells were pelleted and frozen at −20°C. Thawed pellets were resuspended in 50mL of 20 mM Tris-Cl, pH 8.0. One tablet of Complete Protease Inhibitor (Roche, Indianapolis, IN, USA) and 40µl of 50mM phenylmethylsulfonylfluoride (PMSF) were added to inhibit proteolysis. One milliliter of a 25 mg/mL lysozyme stock solution (Acros Organics/Fisher Chemicals, Fairlawn, NJ, USA) was added to the suspension while stirring on ice for 30 minutes. One milliliter of a 100 mg/mL deoxycholic acid stock solution was added and kept stirring on ice for 30 minutes. Subsequently, 2000 U of DNase (Sigma, St Louis, MO, USA) were added to the suspension and heated at 37°C for 15 minutes and then at 23°C for another 15 minutes. The sample was then sonicated 10 minutes, and centrifuged at 17,500 rpm for 30 minutes. Two milliliters of 5% polyethylimine and 0.8 mL of 1 M dithiothreitol (DTT) were added to the supernatant and stirred at 22°C for 10 minutes. The solution was centrifuged at 17,500 rpm for 10 minutes, and then filtered through a 0.22-µm syringe filter. The filtered lysate was loaded onto a pre-equilibrated XK 50/20 column filled with 200 mL of Q Sepharose Fast Flow resin (Amersham Biosciences, Piscataway, NJ, USA). The pre-equilibration buffer was 20 mM Tris-Cl, pH 8.0. Elution fractions were collected over a 3-column volume linear gradient of 0 to 2 M NaCl in 20 mM Tris-Cl, pH 8.0. Elution was monitored at 280 nm, and fractions with absorbance peaks were collected and analyzed by SDS-PAGE. Fractions containing αB crystallin (20kDa) were combined and concentrated to a final volume of 4 mL. A pre-equilibrated Superdex 200 HR 10/30 (Amersham Biosciences) was loaded with 0.5 mL of the filtered concentrated samples. Elution fractions were collected over a 1-column volume of 20 mM Tris-Cl, pH 8.0. Elution fractions were analyzed by SDS-PAGE and pure αB crystallin fractions were pooled. Protein concentration was determined using the BCA protein assay kit (Pierce, Rockford, IL).

### Microtubule assembly assay

The effect of selected molar ratios of αB crystallin∶tubulin on the assembly of tubulin into microtubules *in vitro* was evaluated using the Microtubule stabilization/Destabilization Assay kit (Cytoskeleton; Denver, CO) as described previously [15]. Bovine brain tubulin was dissolved to 200 µM in 80 mM PIPES, 2 mM MgCl2, 0.5 mM EGTA, 10 µM DAPI, 1 mM GTP pH 6.9. 8.5 µl of the tubulin was mixed with 20 µl of 80 mM PIPES, 2 mM MgCl_2_, 0.5 mM EGTA, 7.4 µM DAPI, 16% Glycerol, 1.1 mM GTP pH 6.9 and 25µl of various concentrations of αB crystallin in 20mM Tris-Cl pH 8.0, or tris buffer only. The final tubulin concentration was 34.0µM and final αB crystallin concentrations were 3.40, 4.25, 5.68, 8.50, 17.0, 34.0, 68.0, 136, 204, 272, and 340µM. Microtubule assembly was monitored by measuring the fluorescence of DAPI, (Excitation of DAPI λ = 355nm , emission λ = 460nm) a molecule whose emission fluorescence at λ = 460nm is enhanced 8-fold when it is incorporated into assembled microtubules [41]. Fluorescence of samples were continuously read on a Perkin Elmer Victor3 V fluorescence plate reader (Excitation λ = 355 nm, Emission λ = 460 nm) at 37°C for 45 minutes.

### Aggregation assays

The effect of αB crystallin on the thermal aggregation of tubulin and alcohol dehydrogenase (ADH) was evaluated using ultra-violet spectroscopy. For the tubulin assays 30µl of various concentrations of αB crystallin and 4.25µl of 200µM tubulin were added to 30µl of 80mM PIPES, 2mM MgCl_2_, 0.5mM EGTA, pH 6.9. Samples were heated at 52°C for 60 minutes. The final concentration of tubulin was 17.0µM and the final concentrations of αB crystallin were 2.21, 4.25, 17.0, 68.0µM. Absorbance at λ = 340 nm was measured continuously for 60 minutes using a Pharmacia Biotech Ultrospec 3000. Aggregation assays were conducted in the absence of GTP and glycerol which can induce the assembly of microtubules.

For the ADH assays 10µl of 100µM ADH and 40µl of various concentrations of αB crystallin were added to 150µl of phosphate buffered saline (PBS), pH 7.0. The final concentration of ADH was 5.00µM and the final concentrations of αB crystallin were 0.65, 1.25, 5.00, 20.0µM. Samples were heated at 52°C for 60 minutes. Absorbance at λ = 340 nm was measured continuously for 60 minutes using a Pharmacia Biotech Ultrospec 3000.

### Size exclusion chromatography (SEC)

The interaction between non-polymerized tubulin and human αB crystallin was determined using a Biosep SEC-S4000 column with a molecular weight range of 15–2000 kDa (Phenomenex, Torrance, CA, USA) and an AKTA FPLC Purifier (Amersham Biosciences). Samples were made containing 128µM αB crystallin and/or 16µM tubulin in a final buffer of 20mM tris-HCl, 160µM MgCl_2_, 40µM EGTA, pH 8.0. Mixtures were heated at 37°C for 30 minutes and cooled at 4°C for 10 minutes. 60µL samples were loaded on a pre-equilibrated column and eluted at a flow rate of 1.0 mL/minute in 20mM tris-Cl, pH 8.0 at 4°C. Peaks were recorded at 280 nm and analyzed using Unicorn 4.12 (GE Healthcare). Fractions containing absorbance peaks were collected, concentrated (∼5×) using Vivaspin 6 mL concentrators (Satorius, Goettingen, Germany) and run on SDS-PAGE and stained with Coomassie Brilliant Blue. Molecular-weight protein calibration kits (Amersham Biosciences) were used to calibrate the column. Calibration proteins albumin (67 kDa), aldolase (146 kDa), catalase (226 kDa), thyroglobulin (699 kDa), and blue dextran (2000 kDa) eluted with retention times of 11.15, 9.86, 9.49, 7.88, and 6.43 ml, respectively.

### Sequence alignment and modeling

The sequences of human αB crystallin and other small-heat shock proteins were compared with the sequences of various tubulin proteins for homology using the dot-matrix alignment program ‘Dotter’ [42]. The αB crystallin homologous regions and previously identified intra-microtubule contacts were mapped to the crystal structure of tubulin [43], [44].

The human αB crystallin homology model was computed using the wheat sHSP16.9 X-ray crystal structure as described previously [36], [37].The Cα root mean square deviation between the superimposed model of human αB crystallin and the crystal structure of wheat sHSP16.9 was 3.25Å. The model for the twenty-four subunit oligomer of human αB crystallin was computed using coordinates of the Methanococcus jannaschii sHSP16.5 twenty-four subunit crystal structure described previously. The overall secondary and tertiary structure of the homology model of human αB crystallin is in close agreement with solid state NMR and crystallographic data on the αB crystallin core-domain. [38], [45].

## Results

### Assembly of microtubules is a nonlinear function of the molar ratio of αB crystallin∶tubulin

**Figure 1:**
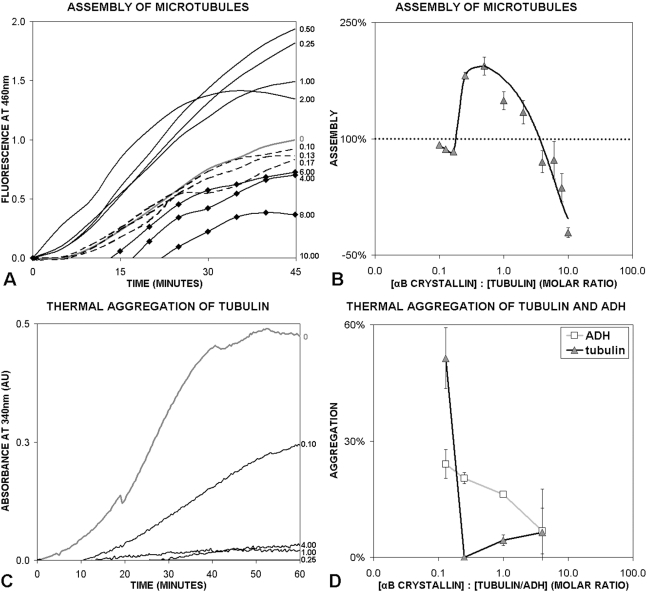
Effects of αB crystallin on microtubule assembly and tubulin aggregation. (A) Microtubule assembly was measured using the DAPI binding assay. In the absence of αB crystallin 34µM tubulin assembled to a fluorescence value (left Y-axis) of 1.0 (grey line, right axis). At molar ratios of αB crystallin∶tubulin (right Y-axis) of 0 to 0.17 (dashed lines) very little effect on microtubule assembly was observed. At molar ratios of αB crystallin∶tubulin between 0.25 and 2.0 (solid black lines), microtubule assembly increased. At molar ratios >2.0 (diamonds), microtubule assembly decreased. (B) Effects of αB crystallin on microtubule assembly at 45 minutes. In the absence of αB crystallin 34µM tubulin assembled to a value of 100% (horizontal dotted line). With increasing molar ratios of αB crystallin∶tubulin, microtubule assembly increased to a maximum approximately two fold greater than in the assembly in the absence of αB crystallin and then decreased at higher molar ratios. The maximum effect was observed at a molar ratio of approximately 0.5. The data were non-linear and the best fit of the data was to a parabola (R^2^ = 0.92). (C) Thermal aggregation of tubulin in the presence and absence of αB crystallin at 52°C. The protective effect of αB crystallin on the thermal aggregation of 17µM tubulin was maximal at a molar ratio αB crystallin∶tubulin of 0.25 (right axis) and decreased at molar ratios above and below 0.25. (D) Comparison of the protective effect of αB crystallin on ADH and tubulin aggregation after 60 minutes at 52°C. The protective effect of αB crystallin on thermal unfolding and aggregation of ADH (grey line, squares) was a linear function of the molar ratio of αB crystallin∶ADH, with the amount of aggregation decreasing as molar ratio increased from 0.10 to 4.0. In contrast, the thermal aggregation of tubulin at 52°C reached a minimum at the molar ratio (αB crystallin∶tubulin) of 0.25 before increasing at molar ratios >0.25(black line, triangles). In the absence of αB crystallin, tubulin and ADH aggregated to a normalized value of 100%. Experiments were conducted using fixed concentrations of ADH (5µM) or tubulin (17µM) and increasing concentrations of αB crystallin.

To quantify the effects of αB crystallin on the assembly and disassembly of microtubules, 34 µM of tubulin was incubated with increasing concentrations of αB crystallin (Fig 1A). At the lowest molar ratios of αB crystallin∶tubulin, no measurable effect on microtubule assembly was observed. Increasing the molar ratio of αB crystallin∶tubulin increased microtubule assembly to a maximum of 2-fold over assembly in the absence of αB crystallin (Fig 1B). At high molar ratios of αB crystallin∶tubulin microtubule assembly decreased. The effect on assembly of microtubules was minimal at molar ratios of αB crystallin∶tubulin less than 0.25 and greater than 2.0. At molar ratios between 0.25 and 2.0, the amount of microtubules formed was 35–94% higher than with tubulin alone with the maximum assembly observed at a molar ratio of 0.50. No microtubules were formed at a molar ratio of αB crystallin∶tubulin of 10. The regulation of microtubule assembly by αB crystallin was a nonlinear function of the ratio of αB crystallin to tubulin.

### Protection against tubulin aggregation is a nonlinear function of the molar ratio of αB crystallin∶tubulin

The effects of αB crystallin on the thermal aggregation of ADH and tubulin were compared using ultraviolet spectroscopy (figure 1C,D). Protection against thermal aggregation of ADH increased linearly with the concentration of αB crystallin. In contrast, the protective effect of αB crystallin on tubulin aggregation was nonlinear. Maximum protection against tubulin aggregation was observed at a molar ratio of αB crystallin∶tubulin of 0.25. The protective effect against aggregation of tubulin and ADH was nearly the same at the molar ratio of αB crystallin∶tubulin of 4.0, the highest molar ratio investigated. It was noted that maximum effect of αB crystallin on tubulin assembly and on aggregation occurred at approximately the same molar ratio of αB crystallin∶tubulin, between 0.25 and 0.50. The results were consistent with the hypothesis that common sequences in αB crystallin are responsible for interactions that influence subunit-subunit interactions during tubulin assembly and the protective effects of αB crystallin on tubulin aggregation.

### Model for interactive domains on human αB crystalline

**Figure 2:**
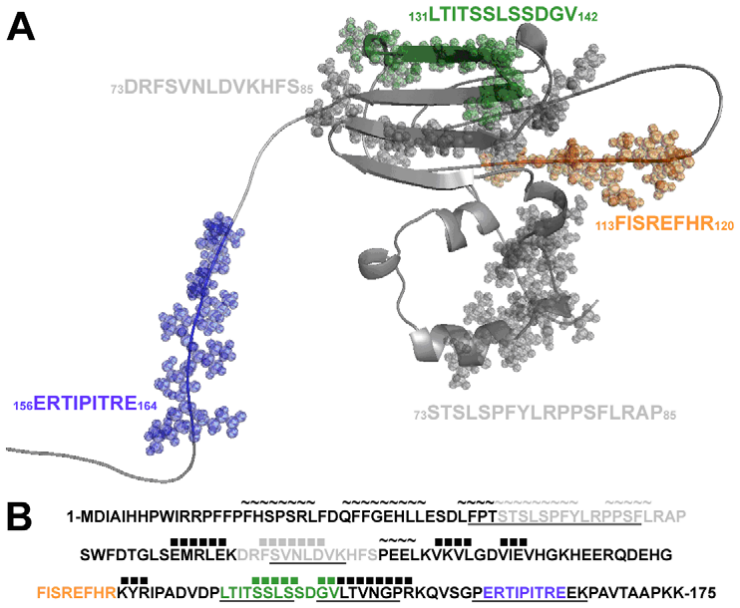
Interactive sequences in the 3D molecular model for αB crystallin. (A) The interactive sequences corresponding to the bioactive peptides in table 1 are represented with space filling spheres in the 3D homology model of human αB crystallin. The microtubule interacting sequences ERTIPITRE (blue) in the C-terminus and LTITSSLSSDGV (green) in the β8 strand of the core domain promoted microtubule assembly, while the sequence FISREFHR (orange) in the core domain loop inhibited microtubule assembly [15]. (B) In the primary sequence of human αB crystallin the grey letters indicate interactive sequences with no interaction with tubulin and the colors indicate microtubule interactive sequences. The secondary structure, α helix (∼) and β strand (▪), is indicated above the primary sequence. Underlined sequences are sites of subunit-subunit interaction [37]. The sequence FISREFHR (orange), which was responsible for inhibiting microtubule assembly, was not involved in the interactions between αB crystallin subunits during complex assembly, while the two sequences responsible for promoting microtubule assembly, ERTIPITRE (blue) and LTITSSLSSDGV (green), were sites of subunit-subunit interaction.

A 3D model of the interactive sequences in αB crystallin is in figure 2. The important interactive sequences were identified as _113_FISREFHR_120_ in the loop of the core α-crystallin domain of αB crystallin, _131_LTITSSLSSDGVL_143_ in the β8 strand of the core α-crystallin domain, and _156_ERTIPITRE_164_ in the C-terminus. Surface exposure of the interactive sequences is expected for the functional effects of αB crystallin on microtubule assembly which can only occur on the surface of dissociated subunits of αB crystallin or tubulin.

### αB crystallin and tubulin form large complexes under non-assembly conditions

**Figure 3:**
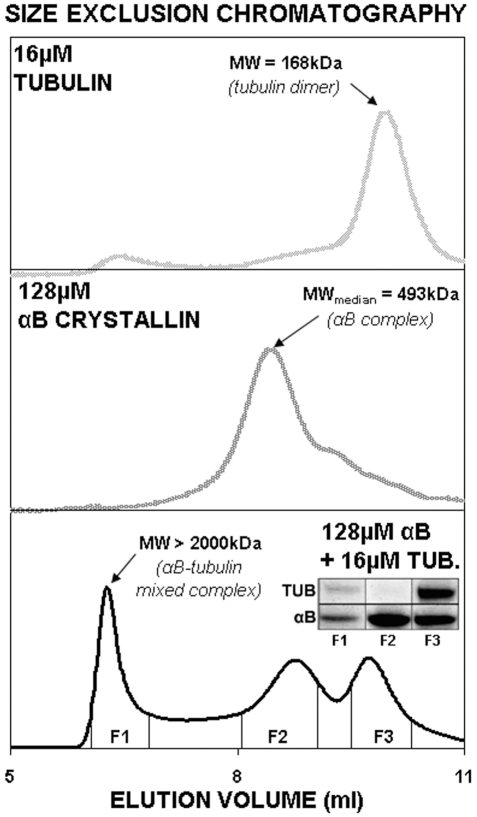
Interactions between tubulin and αB crystallin. The top panel is the SEC profile of 16µM tubulin containing a broad peak with a maximum at 9.96 ml, measured by absorbance at 280nm. The apparent molecular weight (168kDa) is a little larger than the expected value for a tubulin dimer (110kDa). In the middle panel the peak for 128µM αB crystallin eluted at 8.46 ml. The apparent median molecular weight (493kDa) corresponds to an αB crystallin 24-mer, as expected. The bottom panel is the elution profile for a solution containing 280µM αB crystallin and 16µM tubulin which is an 8∶1 molar ratio of αB crystallin∶tubulin. Two peaks were seen at approximately 9.77 and 8.78 ml, corresponding to tubulin and αB crystallin and a new peak at 6.27 ml corresponding to a mass greater than 2000kDa (the exclusion limit of the column). SDS PAGE determined that the new high molecular weight peak (inset F1) contained both tubulin (55kDa monomer) and αB crystallin (20kDa monomer). The F2 peak is unbound αB crystallin. The F3 peak contained αB crystallin and tubulin because of the interaction between αB crystallin and unassembled tubulin subunits. The results confirmed the interaction between subunits of tubulin and αB crystallin can result in formation of very large tubulin - αB crystallin heteromeric complexes.

Size exclusion chromatography (SEC) was used to evaluate the interactions between αB crystallin and unassembled tubulin responsible for formation of large complexes (Fig 3). The SEC elution profile for tubulin alone was a broad peak with a maximum at approximately 9.96 ml corresponding to an apparent molecular weight of 168kDa, somewhat larger than the calculated weight of a tubulin dimer of 110kDa. The SEC profile for αB crystallin alone was a broad peak with a maximum at approximately 8.46 ml with an apparent molecular weight of 493kDa which is consistent with formation of a polydisperse complex having a mean size of 24 subunits. The peaks are broad and overlap because of the polydisperse and dynamic nature of the tubulin filaments and αB crystallin complexes which are known to vary greatly in size. The mixture of tubulin and αB crystallin contained a large elution peak at 6.27ml and separate elution peaks for αB crystallin at approximately 8.78 ml and tubulin 9.77 ml. SDS-PAGE analysis determined that the peak at 8.78ml consisted of αB crystallin only because of the eight fold molar excess and the peak at 9.77ml consisted of both tubulin and αB crystallin. The presence of αB crystallin in the fraction eluting at 9.77ml was due to the interaction of subunits of αB crystallin with subunits of tubulin which can form the polydisperse complexes. The peak at 6.27ml corresponded to an apparent molecular weight >2000 kDa and SDS-PAGE analysis determined that the peak at 6.27ml was composed of both tubulin and the high molecular weight form of αB crystallin. The background absorbance between the peaks is consistent with the polydispersity in the sizes of tubulin - αB crystallin mixed complexes. In the absence of glycerol or GTP, and at a temperature of 4°C microtubule formation was not observed, indicating that the large complexes eluting at 6.27ml (fraction 1, F1) observed in SEC were not microtubules. Taken together, the SEC and SDS-PAGE results were consistent with interactions between subunits of tubulin and αB crystallin and the formation of mixed αB crystallin and tubulin complexes of varying size ranging from approximately 500 kDa to greater than 2000kDa.

### Tubulin contains interactive sequences found in αB crystalline

**Figure 4:**
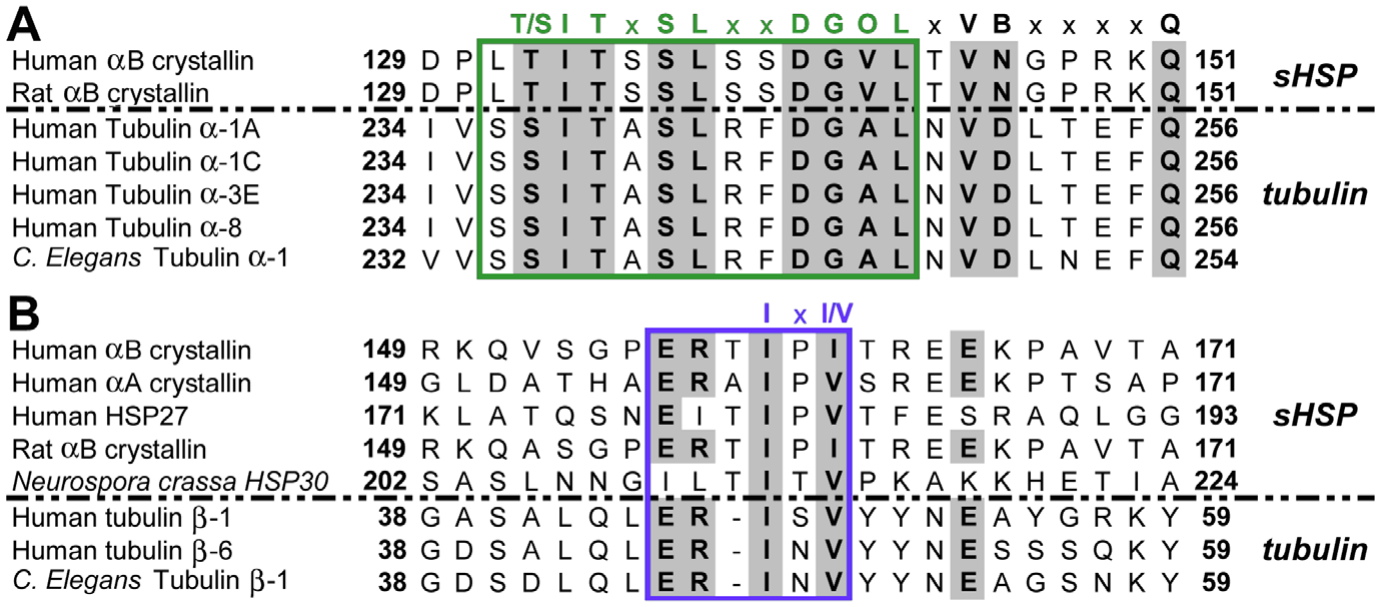
Common interactive sequences in sHSPs and tubulins. (A) Comparison of the amino-acid sequences of human and rat αB crystallin, human tubulin α-1A, α-1c, α-3E, α-8, and C. elegans tubulin α-1. The region containing the β8-strand of αB crystallin (129–151) shares homology with the region 234–256 in human α tubulin and 232–254 in C. elegans tubulin α-1. The β8 region of αB crystallin is within the box and the residues highlighted in grey are conserved. This sequence is an αB crystallin subunit-subunit interactive site. (B) Comparison of the amino-acid sequences of human αB crystallin, αA crystallin, HSP27, rat αB crystallin, Neurospora crassa HSP30, human tubulin β-1, β-6, and C. elegans tubulin β-1. The region 47–49 in the β tubulin sequences is homologous to the conserved I-X-I/V motif in the small heat-shock proteins and is an interactive sequence involved in the assembly of the sHSP complex(box). This sequence is an αB crystallin subunit-subunit interactive site.

**Figure 5:**
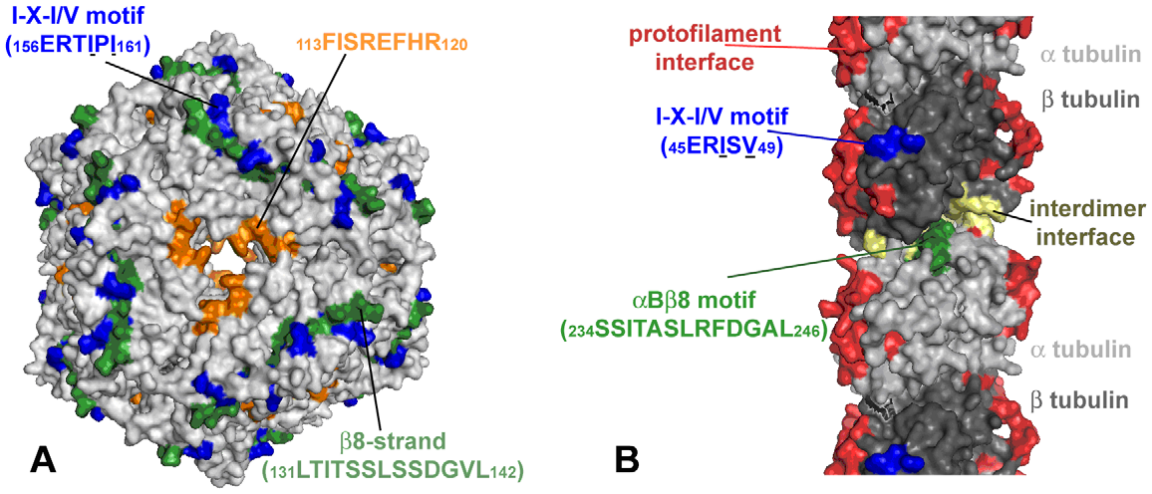
Exposure of interactive sequences in the 3D molecular models for αB crystallin complex and the tubulin protofilament. (A) In the 3D model for the spherical complex of human αB crystallin, the microtubule interactive and subunit-subunit interactive sequences _156_ERTIPI_161_ (blue) and _131_LTITSSLSSDGVL_142_ (green) in the β8-strand are partially buried. The tubulin interactive sequence, _113_FISREFHR_120_ (orange), which is not a subunit-subunit interactive site surrounds a window within the complex. The dynamic equilibrium between subunits and complexes regulates access to the interactive surface domains on subunits of αB crystallin. (B) The 3D model of a microtubule protofilament contains an interdimer interface (yellow) where protofilaments are organized and the protofilament interface that is used in the formation of microtubules from protofilaments (red). The regions homologous to the I-X-I/V (blue) and β8-motifs (green) of αB crystallin are found in the lumen of the hollow microtubule. These corresponding sequences in αB crystallin are subunit-subunit interactive sites, meaning that these homologous sequences in tubulin are potential sites for interactions with αB crystallin subunits. The molecular models for the structure of the αB crystallin complex and the assembled microtubules are consistent with the results in figures 2 and 3 suggesting that interactions between subunits can shift the dynamic equilibria to favor the assembly of microtubules or αB crystallin complexes (see model figure 6). Isoforms of the microtubule associated protein tau bind the luminal side of microtubules near the interdimer interface, similar to a site for the predicted interaction with αB crystallin.

Homologous sequences in αB crystallin, other sHSPs and tubulin (figures 4 and 5) were identified. The sequence 129–151 from both human and rat αB crystallin was found to be homologous with the sequence 234–256 from human α tubulin and 232–254 from *C. elegans* α-1 tubulin. The human αB crystallin β8-strand, _131_LTITSSLSSDGVL_143_, identified as a microtubule interactive site, shares homology with a human α tubulin sequence, _234_SSITASLRFDGAL_246_. A short but important region of sequence homology between human αB crystallin and β tubulin was identified as the microtubule interactive region _156_ERTIPI_161_ in human αB crystallin which was homologous to the region _45_ERISV_49_ in human β-1 tubulin and _45_ERINV_52_ from human β-6 tubulin. This sequence in αB crystallin contains the critical I-X-I/V motif which is involved in αB crystallin function and complex assembly. Both the αBβ8- and the I-X-I/V homology motifs are found on the luminal side of the tubulin dimer, near the inter-dimer interface, where two dimers bind together (Fig 5). The interactive sequences identified in the homology models (Figs 4&5) are the basis for the interactions between tubulin and αB crystallin subunits in the regulation of tubulin assembly by αB crystallin. The common interactive domains on tubulin and αB crystallin are sites for interaction between the subunits of both systems.

## Discussion

**Figure 6:**
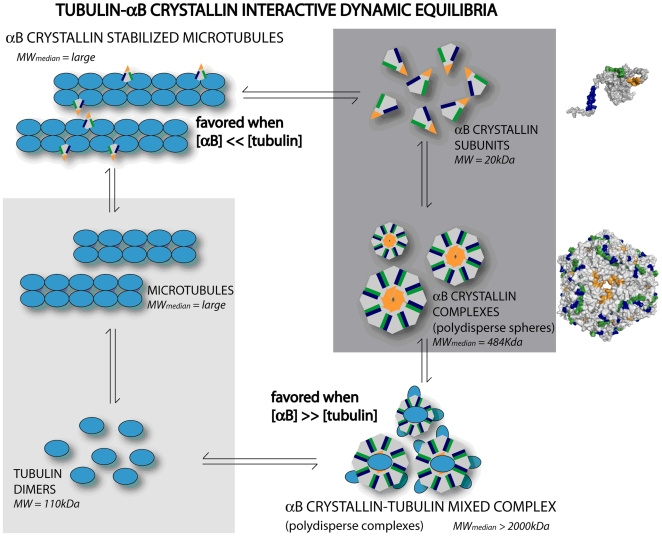
Model for linked dynamic equilibria between tubulin and αB crystallin. The shaded inserts model two dynamic equilibria for tubulin and αB crystallin. Tubulin subunits are in dynamic equilibrium with microtubules in the absence of αB crystallin (lower left insert) and αB crystallin subunits are in dynamic equilibrium (dynamic subunit exchange) with polydisperse spherical complexes in the absence of tubulin (upper right insert). In the proposed model for regulation of tubulin assembly by αB crystallin, subunits of tubulin can interact with subunits of αB crystallin and the molar ratio of αB crystallin∶tubulin can influence the two dynamic equilibria to regulate the assembly of microtubules. The effect of αB crystallin on microtubule assembly depends on the molar ratio of αB crystallin∶tubulin (see fig 1). When the molar ratio of αB crystallin∶tubulin is small, microtubule assembly is favored and αB crystallin monomers stabilize assembled microtubules (upper left). When the molar ratio of αB crystallin∶tubulin is large, assembly of mixed αB crystallin-tubulin complexes is favored, decreasing the tubulin available for assembly into microtubules (lower right). Under the conditions used in these studies, maximum tubulin assembly was observed at a molar ratio of approximately 0.5 αB crystallin∶tubulin which is 2 molecules of tubulin for each molecule of αB crystallin stabilized microtubule formation (upper left) . In cells, subunits of tubulin can interact with subunits of αB crystallin and the molar ratio of αB crystallin∶tubulin can influence the two dynamic equilibria to regulate the assembly of microtubules (space-filled models of αB crystallin and the αB complex are shown on the right).

The results of the current study identified sequences in αB crystallin and tubulin that account for the observed effects of αB crystallin on enhancement or inhibition of microtubule assembly [15]. The effect of αB crystallin on tubulin assembly or thermal aggregation is a nonlinear function of the molar ratio of αB crystallin∶tubulin. At large molar ratios of αB crystallin to tubulin, microtubule assembly was inhibited. The interactions between αB crystallin and tubulin were demonstrated by SEC and SDS-PAGE, which recorded the formation of large heterogeneous co-complexes under non-assembly conditions for microtubules. The mechanisms for microtubule assembly and complex assembly of αB crystallin are both dynamic equilibria. The model for the action of αB crystallin on tubulin assembly in vitro or in vivo is a linked mechanism between the two dynamic equilibria (Fig 6).

The mechanism for linked dynamic equilibria regulating the self assembly of αB crystallin or tubulin subunits is based on the common interactive domains on the surfaces of tubulin and αB crystallin molecules. Two interactive motifs in αB crystallin, _131_LTITSSLSSDGV_142_ and _156_ERTIPITRE_164_, have homologous motifs in the primary sequence of tubulin. The sequence _131_LTITSSLSSDGV_142_ in the β8 strand of αB crystallin is important for complex formation, binding of unfolding proteins, and interactions with filament proteins [22], [37], [46] and the C-terminal sequence _156_ERTIPITRE_164_ contains the I-X-I/V motif, which is critical for preventing aggregation of unfolding proteins and for complex formation of αB crystallin [47], [48]. in the presence of the synthetic peptides, _131_LTITSSLSSDGV_142_ and _156_ERTIPITRE_164_, microtubule assembly was stabilized and promoted [15]. In the crystal structures of homologous small-heat shock proteins, the residues in the β8 strand of one αB crystallin subunit can bind the residues in and around the I-X-I/V motif in an adjacent subunit [35], [36].

In tubulin, sequences homologous to the interactive domains in αB crystallin were identified which was consistent with the model for linked dynamic equilibria between tubulin and αB crystallin. The sequence _234_SSITASLRFDGAL_246_ in tubulin resembles _131_LTITSSLSSDGVL_143_ in the β8-strand in the core domain of αB crystallin and the sequence _45_ERISV_49_ in β tubulin resembles _156_ERTIPI_161_ in the C-terminus of αB crystallin which contains the I-X-I/V interactive motif. In tubulin, the sequences are on the surface of the luminal side of the microtubule near a taxol binding site at the interface between two tubulin dimers [43], [44]. It is expected that interactive domains on the surface of αB crystallin can be occupied by tubulin subunits when the ratio of αB crystallin∶tubulin is small and αB crystallin is unavailable to interact with other αB crystallin subunits to form a complex. At large ratios of αB crystallin∶tubulin, the interactive domains not involved in subunit – subunit interactions are occupied by tubulin. Low resolution electron cryomicroscopy identified a binding site for the microtubule-associated protein tau near the taxol binding site at the interface between two tubulin dimers [39]. Interestingly, a 3-repeat isoform of tau inhibits microtubule assembly at the lowest molar ratios of tau∶tubulin (1∶55-1∶45), but promotes microtubule assembly higher molar ratios (>1∶38) [49] suggesting a regulatory function for the tau site in tubulin assembly. A similar interaction with the αB crystallin subunits at the tau site on the luminal surface could influence tubulin assembly. In our model the I-X-I/V motif on the surface of one tubulin dimer interacts with the β8-motif on the surface of an αB crystallin subunit and the αBβ8-motif on an adjacent tubulin dimer interacts with the I-X-I/V motif of αB crystallin. The interactive sequences identified using sequence analysis account for the observed link between the dynamic equilibrium for the assembly of microtubules and the dynamic equilibrium for assembly of αB crystallin complexes. While the results did not show that sHSP substitutes for tau, the tau regulatory site may be used by αB crystallin to regulate MT assembly which suggests the potential importance of the interactive sequences in the β8 strand and in the C-terminus in the dynamic mechanism for the function of human αB crystallin.

The results supporting the importance of the dynamic mechanism in the function of αB crystallin are: (a) With increasing molar ratios of αB crystallin∶tubulin, the dynamic equilibrium favoring tubulin assembly was first promoted and then inhibited [15]. (b) Protein pin arrays identified the sequence _113_FISREFHR_120_ in the core domain loop as an interactive site for unfolding proteins and filaments in human αB crystallin [16], [22], [37]. (c) In the crystal structures of other sHSPs, the sequence _113_FISREFHR_120_ was not involved in subunit-subunit interactions and was exposed on the surface of the sHSP complex [35], [36] suggesting that an accessible _113_FISREFHR_120_ sequence for binding target proteins , such as tubulin, even when αB crystallin is in a complex. The structural data are consistent with biochemical and computational data indicating that the _113_FISREFHR_120_ sequence is not involved in subunit-subunit interactions and is available for interaction with target proteins [15], [37], [50]. Recent crystallographic and NMR studies of truncated human αB crystallin core-domains suggest that the _113_FISREFHR_120_ sequence may be involved in dimerization [38], [45] through hydrogen bonds of the backbone while the F113, S115, E117, H119 side chains are exposed on the surface of the αB crystallin complex and are available for interactions with tubulin. (d) The SEC results and previous sucrose gradient centrifugation data were consistent with the point that αB crystallin interactions with unassembled tubulin results in large polydisperse complexes [30]. (e) Increasing the molar ratio of αB crystallin∶tubulin increased complex formation. Under conditions favorable for αB crystallin-tubulin mixed complexes, the pool of tubulin subunits decreased and assembly of microtubules was inhibited. The dynamic model for regulation of tubulin assembly by αB crystallin is consistent with published reports on the importance of dynamic subunit exchange and functional activity of αB crystallin [14], [51], [52].

The linked dynamic equilibria can account for the difference between the protective effect of αB crystallin on aggregation of tubulin and ADH (Fig 6). The protective effect of αB crystallin on ADH was linear which is consistent with a continuous and increasing interaction between αB crystallin subunits during the progressive thermal unfolding and aggregation of ADH at high temperature. In contrast, the protective effect of αB crystallin on the aggregation of unfolding tubulin was nonlinear indicating a second factor, the dynamic equilibrium, was important in the protection against tubulin aggregation. It is noted that the maximum protective effect of αB crystallin on tubulin assembly and on tubulin aggregation occurred at approximately the same molar ratio of 0.2 αB crystallin∶tubulin suggesting a similar mechanism for the nonlinear effect of αB crystallin on tubulin aggregation and on tubulin assembly. This mechanism is consistent with a published report in which changes in the ratio of αB crystallin∶tubulin can regulate microtubule dynamics in muscle [28]. We conclude that the effects of αB crystallin on microtubule assembly and aggregation were the result of a unique mechanism linking two dynamic equilibria: one for the assembly of microtubules, and one for the assembly of αB crystallin complexes. Varying the molar ratio of αB crystallin∶tubulin regulated the assembly of microtubules by shifting the equilibria between αB crystallin and tubulin which had important functional consequences for the actions of αB crystallin. In normal cell differentiation and in protection against the early stages of protein unfolding disorders, αB crystallin subunits not only associated with themselves, but also with a soluble pool of tubulin subunits in the cytoplasm. Common interactive domains on αB crystallin for tubulin and αB crystallin subunits regulated quaternary structure and the dynamic assembly of microtubules.

**Table 1:** Effects of the interactive sequences of αB crystallin on microtubule assembly.

Peptide	Residues	location	Microtubule assembly	Subunit-subunit interactions
STSLSPFYLRPPSFLRAP	41–58	N-terminus	NE	Yes
DRFSVNLDVKHFS	73–85	β3 strand	NE	Yes
FISREFHR	113–120	core-domain loop	−	No
LTITSSLSSDGV	131–142	β8 strand	+	Yes
ERTIPITRE	156–164	C-terminus	+	Yes

Column one lists the bioactive peptides based on the interactive sequences in αB crystallin and tested previously on microtubule assembly [15]. Column two lists the corresponding residues in human αB crystallin. Column three lists the location in human αB crystallin of each interactive sequence. Column four lists the effect of each peptide on microtubule assembly; “NE” indicates “no effect”, “−” indicates inhibition and “+” indicates promotion of assembly. Column five lists the involvement of each sequence in subunit-subunit interactions. The two peptides that promote microtubule assembly are sequences for subunit-subunit interactions and the peptide that inhibits microtubule assembly is not. The interactions between tubulin and αB crystallin occur at sites where these sequences are exposed on the surface of the αB crystallin subunit.
